# Effects of spray-dried animal plasma on growth performance, survival, feed utilization, immune responses, and resistance to *Vibrio parahaemolyticus* infection of Pacific white shrimp (*Litopenaeus vannamei*)

**DOI:** 10.1371/journal.pone.0257792

**Published:** 2021-09-24

**Authors:** Niti Chuchird, Tirawat Rairat, Arunothai Keetanon, Putsucha Phansawat, Chi-Chung Chou, Joy Campbell

**Affiliations:** 1 Faculty of Fisheries, Department of Fishery Biology, Kasetsart University, Chatuchark, Bangkok, Thailand; 2 Department of Veterinary Medicine, College of Veterinary Medicine, National Chung Hsing University, Taichung, Taiwan; 3 APC LLC, 2425 SE Oak Tree Court, Ankeny, Iowa, United States of America; Kafrelsheikh University, EGYPT

## Abstract

Spray-dried animal plasma (SDP) in feed for several animal species provides health benefits, but research about use of SDP in shrimp feed is very limited. The objectives of the present study were to investigate the effects of dietary SDP on growth performance, feed utilization, immune responses, and prevention of *Vibrio parahaemolyticus* infection in Pacific white shrimp (*Litopenaeus vannamei*). In Experiment 1, the post-larvae were divided into five groups (four tank/group and 80 shrimp/tank) and fed four times daily diets with porcine SDP at 0, 1.5, 3, 4.5, and 6% of the diet for 45 days. In Experiment 2, the surviving shrimp from Experiment 1 were redistributed into six groups: four SDP groups as in Experiment 1 plus the positive and negative controls (four tank/group and 30 shrimp/tank). They were then challenged with *V*. *parahaemolyticus* by immersion at 10^5^ colony-forming units (CFU)/mL and were fed with the same diets for another 4 days. In Experiment 1, shrimp fed 4.5% or 6% SDP diets had significantly higher body weight, survival rate, and improved feed conversion ratio. The immune parameters (total hemocyte count and phagocytic, phenoloxidase, and superoxide dismutase activities) of the shrimp fed 3–6% SDP diets also showed significant enhancement compared to the control. In Experiment 2, the survival rates of the 3–6% SDP groups were significantly higher than the positive control at day 4 after the immersion challenge. Likewise, the histopathological study revealed milder signs of bacterial infection in the hepatopancreas of the 3–6% SDP groups compared to the challenged positive control and 1.5% SDP groups. In conclusion, shrimp fed diets with SDP, especially at 4.5–6% of the diet, showed significant improvement in overall health conditions and better resistance to *V*. *parahaemolyticus* infection.

## Introduction

In the intensive aquaculture system, high stocking density often increases shrimp stress and makes them more susceptible to infectious diseases, especially with the higher inclusion of vegetable derived proteins replacing fish meal which have lower digestible protein and therefore provide more favorable conditions for specific pathogenic bacterial growth in ponds. In addition, because the prophylactic use of antibiotics in shrimp feed is now restricted or banned in many shrimp producing countries, it is wise to find alternatives for promoting shrimp well-being and reducing severity of disease outbreaks without causing negative impacts on the environment or consumer’s health [1,2].

Spray-dried animal plasma (SDP) is a protein-rich animal blood by-product obtained from abattoirs. Industrial production of SDP involves the separation of the plasma from blood cells by centrifugation, concentration by vacuum evaporation or filtration, and spray-drying [3,4]. SDP is usually prepared from either pig (spray-dried porcine plasma; SDPP) or cattle blood (spray-dried bovine plasma; SDBP). It contains a diverse biological component including immunoglobulins (Ig), albumin, peptides, enzymes, transferrin, fibrinogen, and growth factors. As an animal feed protein source, SDP has long been proven to promote animal health [4,5]. Regarding the biosafety concern of SDP products, it should be noted that only the blood from healthy pigs was collected, and the spray-drying process can effectively eliminate any potential bacterial and viral pathogens [6]. Therefore, the risk of disease transmission to human consumers as a result of eating SDP-fed shrimps was unlikely.

The health-promoting property of SDP is well-known in farmed animal production and the scientific evidence supporting its application, most notably in weaned pig feeds, is extensive. This includes enhancing growth performance, nutrient utilization, and feed intake, suppressing inflammation and disease incidence, and influencing gut microbiota in a positive manner [3,4,6]. Supplementation of SDP in the feed at 5–8% of the diet (50–80 g/kg diet) for 14–35 days was reported to significantly enhance the body weight and feed intake of weaned pigs with or without bacterial challenge [7–11]. Thus, it has been proposed for use as a growth promoter in the livestock industry. The health benefits of SDP regarding growth and feed utilization have been reported in broilers as well [5] usually about 1–2% of the diet, compared to higher levels reported in pig studies [12–14].

Due to the rich protein content, SDP has been evaluated for application in aquatic feeds as a partial replacement of fish meal with promising results. Spray-dried blood products were highly digestible in rainbow trout (*Oncorhynchus mykiss*), and the digestibility of SDP was significantly higher than that of the spray-dried blood cells and spray-dried whole blood [15]. The digestibility of SDP in rainbow trout was also greater than that of the spent hen meal, poultry by-product meal, and feather meal [16]. Likewise, the apparent digestibility coefficients for dry matter, crude protein, crude lipid, and gross energy of SDP in Pacific white shrimp (*Litopenaeus vannamei*) were in the range of 71–92% which was comparable to those of the fish meal and significantly greater than other animal proteins including shrimp by-product meal, meat and bone meal, and poultry meat meal [17]. However, the potential benefits of SDP in promoting aquatic animal health have just recently been reported. For instance, gilthead seabream (*Sparus aurata*) that were fed 3% porcine SDP for 60–95 days showed significantly higher body weight compared to the control groups [18,19]. Feeding Nile tilapia (*Oreochromis niloticus*) with 4.97–6.63% porcine SDP for 60 days resulted in improved body weight, feed intake, and feed conversion ratio (FCR) [20]. In another study, the fish meal component in the diet could be completely replaced by bovine plasma protein concentrate without harming the growth performance of pacu (*Piaractus mesopotamicus*) [21]. Other advantages of SDP reported in the fish studies include enhanced serum innate immunity [18] and improved intestinal morphology [20]. These studies demonstrated that the beneficial effects of SDP in fish diets were in general similar to results observed in terrestrial animals.

The effects of SDP on shrimp health is not well understood. To the best of our knowledge, information regarding the potential advantages of SDP in shrimp culture was only limited to one exploratory study published in a non-peer-reviewed source which concluded that diets supplemented with 1–3% SDP was able to increase the growth and survival rate of black tiger shrimp (*Penaeus monodon*) in laboratory conditions and that 6% SDP could improve the body weight and survival rate of Pacific white shrimp in a field trial [22].

SDP should be considered as a promising alternative because of its beneficial effects on mammalian and fish health have been reported in many publications. The current study aimed to investigate the health-promoting effects of porcine SDP in diets of Pacific white shrimp. The study was divided into two experiments. In Experiment 1, the effects of SDP on growth, survival, feed utilization, and immune responses were evaluated in healthy shrimp. In Experiment 2, the influences of SDP on resistance to *Vibrio parahaemolyticus* was assessed after the experimental infection challenge. The findings of the present study were expected to provide scientific evidence in support of the application of SDP in feed for Pacific white shrimp farming for sustainable aquaculture.

## Materials and methods

### Experiment 1: Effects of SDP on growth performance, survival, feed utilization, and immune responses of healthy shrimp

#### Preparation of the experimental diets

Porcine blood was collected from healthy pigs at abattoirs, mixed with anticoagulants to prevent blood clotting, centrifuged to separate the plasma from the blood cells, then concentrated and spray-dried at high temperature (80°C) into a powder containing < 8% moisture and > 75% crude protein. Five experimental diets were formulated with different concentrations of SDP (AP 820, APC Europe, Granollers, Spain): 0 (control), 1.5, 3, 4.5, and 6% SDP (or 0, 15, 30, 45, and 60 g/kg diet). The SDP was incorporated into the basal diet at the expense of soybean meal and amounts were adjusted using wheat flour. The ingredients and proximate composition of the basal diet are shown in Table 1. The experimental diets were prepared by mixing the desired amount of SDP with the other feed ingredients and subjecting the mash feed to a pelleting process by a feed pelleting machine.

**Table 1:** Ingredients and proximate composition (%) of the experimental diets.

Ingredients (%)	Control	1.5% SDP	3% SDP	4.5% SDP	6% SDP
Fish meal	7.63	7.63	7.63	7.63	7.63
Soybean meal	25.00	22.15	19.30	16.45	13.60
Spray-dried porcine plasma	0.00	1.50	3.00	4.50	6.00
Poultry meal	22.25	22.25	22.25	22.25	22.25
Wheat gluten	1.25	1.25	1.25	1.25	1.25
Corn protein concentration	4.50	4.50	4.50	4.50	4.50
Wheat flour	25.00	26.35	27.70	29.05	30.40
Premix*	2.10	2.10	2.10	2.10	2.10
Preservative	4.53	4.53	4.53	4.53	4.53
Attractant	7.75	7.75	7.75	7.75	7.75
**Proximate composition (%)**					
Ash	15.44	14.37	14.49	13.74	13.11
Carbohydrate	28.36	29.58	29.77	30.82	31.70
Lipid	8.00	7.64	7.60	7.23	6.84
Moisture	10.12	10.30	9.70	9.68	9.66
Protein	38.08	38.11	38.44	38.53	38.69

Note:

*Premix composition (per kg) as follows: vitamin A 6,700,000 IU, vitamin D 1,350,000 IU, vitamin E 67 g, vitamin K3 3.4 g, vitamin B1 6.7 g, vitamin B2 10 g, vitamin B6 8 g, vitamin B12 13.5 g, niacin 53 g, pantothenic 26.5 g, folic acid 3.3 g, and biotin 335 g.

#### Experimental animals

Three thousand Pacific white shrimp (*Litopenaeus vannamei*) postlarvae 9 (PL-9) from a commercial shrimp hatchery in Chachoengsao Province, Thailand were transported to the Aquaculture Business Research Center (ABRC) laboratory, Faculty of Fisheries, Kasetsart University, Thailand. They were acclimatized in a 500 L fiberglass tank at 27–29°C and 25 ppt salinity for 3 days until reached the PL-12 stage. Then, 1,600 PL-12 were randomly distributed into 20 500-L fiberglass tanks with 200 L of water (5 treatment groups with 4 replicates) with a stocking density of 80 shrimp/tank which is equivalent to 120 shrimp/m^2^. Water quality parameter analysis was conducted weekly. The dissolved oxygen was measured by YSI PRO 20 (YSI Inc. / Xylem Inc., Yellow Springs, OH, USA) and pH was measured by EcoScan pH 5 (Thermo Fisher Scientific Inc.). Total ammonia and nitrite were analyzed according to American Public Health Association, American Water Works Association, and Water Environment Federation [23]. The water quality parameters of all groups throughout the feeding trial were suitable for marine shrimp culture. Specifically, the pH was about 8.3, dissolved oxygen > 6.5 mg/L, total ammonia < 0.4 mg/L, and nitrite < 0.3 mg/L.

#### Growth and survival study

The shrimp were fed *ad libitum* with one of the five experimental diets four times daily for 45 days. On day 30 and 45 of the feeding trial, 10 shrimp in each tank were randomly selected and weighted using a balance with 2 decimal places and the survival rates were also counted at the same time. The feed intake, feed conversion ratio (FCR, total feed fed divided by body weight gain), feed efficiency (FE, body weight gain divided by total feed fed x 100), and protein efficiency ratio (PER, weight gain per unit of protein fed) were determined at the end of Experiment [24].

#### Immunological study

On day 45, five shrimp from each group were randomly selected for the immunological study which consists of total hemocyte count, phagocytic activity, phenoloxidase activity, and superoxide dismutase (SOD) activity. Hemolymph was collected from each individual shrimp (0.2 mL) with a syringe containing 0.6 mL of anticoagulant (K-199 + 5% l-cysteine).

Total hemocyte count was measured from a mixture of hemolymph-anticoagulant (100 μL) using a hemocytometer under a light microscope and reported as cell/mL.

Phagocytic activity procedure followed the method described by Itami *et al*. [25]. The collected shrimp hemocytes were rinsed with shrimp saline and adjusted to a concentration of 1 × 10^6^ cells/mL. The cell suspension (200 μL) was added into a cover slip and incubated for 20 min; then, washed three times with shrimp saline. Heat-killed yeast (2 mL) was added, incubated for 2 h, washed five times with shrimp saline, and fixed with 100% methanol. The hemocytes on the cover slip were stained with Giemsa stain and mounted with Permount Mounting Medium. Two hundred hemocytes were count and observed for a presence or absence of the phagocytosed yeast cells. Phagocytic activity was defined as the percentage of phagocytic hemocytes per total hemocytes.

Phenoloxidase activity was determined following the method described by Supamattaya *et al*. [26]. The hemolymph-anticoagulant mixture (200 μL) was washed three times with shrimp saline and centrifuged at 1000 rpm, 4°C, for 10 min. The hemocyte lysate (HLS) was prepared from hemocytes in a cacodylate buffer, pH 7.4, using a sonicator at 30 amplitude for 5 s. Then, the suspension was centrifuged at 10,000 rpm, 4°C for 20 min, and the supernatant was collected. The, 200 μL of HLS was mixed with 200 μL of 0.1% trypsin in cacodylate buffer and 200 μL of 4 mg/mL l-dihydroxyphenylalanine (l-DOPA). Enzyme activity was measured as the absorbance of dopachrome at 490 nm. The protein content measurement in the HLS followed the method of Lowry *et al*. [27]. The phenoloxidase activity was presented as the increase in optimum density/min/mg of protein.

Superoxide dismutase (SOD) activity of the blood (20 μL) was analyzed by a SOD Assay Kit (Sigma-Aldrich) following the manufacturer’s instructions.

### Experiment 2: Effects of SDP on resistance to *V*. *parahaemolyticus* infection of shrimp after immersion challenge

#### Immersion challenge with *Vibrio parahaemolyticus*

A total of six experimental groups were studied in Experiment 2. The juvenile shrimp (about 2 g) that had been fed one of the four SDP-supplemented diets from Experiment 1 were redistributed into new 16 500-L fiberglass tanks (four replicates/treatment group and 30 shrimp/tank). The control shrimp from Experiment 1 were randomly divided into two new groups: positive control (with *V*. *parahaemolyticus* challenge) and negative control (without *V*. *parahaemolyticus* challenge), each group consisted of four replicate and 30 shrimp/tank. The animal husbandry and rearing water quality were similar to that of Experiment 1. The shrimp in all groups except the negative control were challenged by immersion with *Vibrio parahaemolyticus* (TISTR 1596) at the final concentration of 10^5^ CFU/ml. The methodology of bacterial preparation was previously described in our recent study [2].

#### Growth and survival study

Three hours after the bacterial inoculation, the shrimp in each tank were provided with one of the five experimental diets four times daily at the satiation rate for an additional 4 days. The survival rate was determined daily, while the weight gain was determined at the end of the study.

#### Histopathological study

Five shrimp were sampled from each group at the end of the trial (day 4 after the immersion challenge). The head of each shrimp was fixed with Davidson’s fixative and processed for histological study to evaluate the histopathological change of the hepatopancreas following the standard protocol [28].

#### Statistical analysis

The difference among treatment groups was analyzed by one-way analysis of variance (ANOVA) followed by Duncan’s multiple range test using IBM SPSS Statistics version 27 software (IBM Corporation, Armonk, NY, USA). Differences were considered statistically significant if *P* < 0.05.

## Results

### Experiment 1: Effects of SDP on growth performance, survival, feed utilization, and immune responses of healthy shrimp

After 45 days of the feeding trial, shrimp fed with 6% SDP groups (3.13 g) had the highest average body weight, followed by the 4.5% (3.06 g) and 3% SDP (2.88 g) which were significantly higher (p < 0.05) than the control and 1.5% SDP groups (Table 2). The same was true for the feed intake even though no statistical analysis was performed in this parameter (Table 2). Similarly, the feed utilization of the 3–6% SDP groups showed a significant improvement (p < 0.05) compared to the control shrimp as indicated by the FCR, FE, and PER (Table 3). Likewise, the highest average survival rate on day 45 was observed in 4.5% and 6% SDP groups (approximately 86%), significantly greater (p < 0.05) than the control group (78%) (Table 4). The shrimp fed 1.5% and 3% SDP showed a slight improvement in terms of their survival rate.

**Table 2:** Effects of SDP on body weight and feed intake.

Treatment group	Body weight (g)		Feed intake (kg)
	Day 30	Day 45	
**Control**	1.10 ± 0.03^a^	2.58 ± 0.02^c^	1.0
**1.5% SDP**	1.14 ± 0.02^b^	2.73 ± 0.12^c^	1.1
**3% SDP**	1.20 ± 0.03^a^	2.88 ± 0.10^b^	1.1
**4.5% SDP**	1.21 ± 0.01^a^	3.06 ± 0.14^a^	1.2
**6% SDP**	1.23 ± 0.04^a^	3.13 ± 0.07^a^	1.2

The data was presented as mean ± SD. Means with different superscripts in a column are significantly different from each other (p < 0.05).

**Table 3:** Effects of SDP on feed conversion ratio (FCR), feed efficiency (FE), and protein efficiency ratio (PER).

Treatment group	FCR	FE	PER
**Control**	1.55 ± 0.02^b^	64.44 ± 0.90^b^	1.66 ± 0.02^c^
**1.5% SDP**	1.54 ± 0.08^b^	64.86 ± 3.38^b^	1.67 ± 0.09^bc^
**3% SDP**	1.45 ± 0.05^a^	69.11 ± 2.59^a^	1.78 ± 0.07^ab^
**4.5% SDP**	1.43 ± 0.08^a^	70.24 ± 3.81^a^	1.81 ± 0.10^a^
**6% SDP**	1.39 ± 0.05^a^	71.95 ± 2.45^a^	1.85 ± 0.06^a^

The data was presented as mean ± SD. Means with different superscripts in a column are significantly different from each other (p < 0.05).

**Table 4:** Effects of SDP on survival rate of the healthy shrimp.

Treatment group	Survival rate (%)		
	Day 20	Day 30	Day 45
**Control**	89.38 ± 5.99^a^	83.13 ± 0.72^b^	78.13 ± 0.72^b^
**1.5% SDP**	86.56 ± 4.61^a^	83.44 ± 3.29^b^	80.31 ± 1.20^ab^
**3% SDP**	89.69 ± 1.57^a^	84.69 ± 3.87^ab^	80.94 ± 0.63^ab^
**4.5% SDP**	88.13 ± 3.31^a^	87.50 ± 2.70^ab^	85.94 ± 2.13^a^
**6% SDP**	92.50 ± 4.45^a^	88.75 ± 4.21^a^	86.25 ± 2.50^a^

The data was presented as mean ± SD. Means with different superscripts in a column are significantly different from each other (p < 0.05).

**Fig 1:**
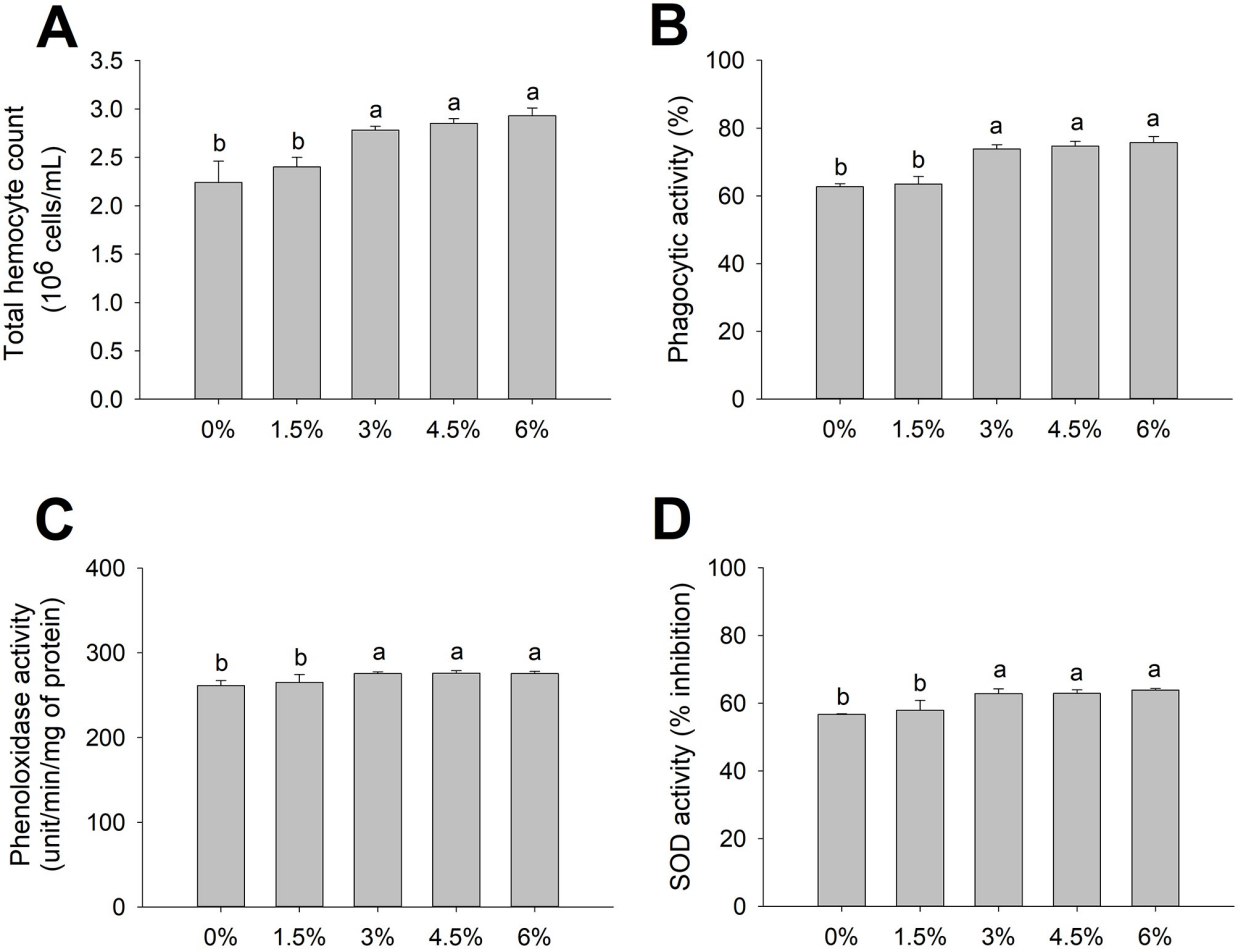
Effects SDP on immune parameters of healthy shrimp. Total hemocyte count (10^6^ cells/mL) (**A**), phagocytic activity (%) (**B**), phenoloxidase activity (unit/min/mg of protein) (**C**), and superoxide dismutase (SOD) activity (% inhibition) (**D**) of the shrimp (*n* = 5) fed the control diet, 1.5%, 3%, 4.5% and 6% SDP on day 45. The data are presented as the mean ± standard deviation. Different letters above the bars indicate significant differences (p < 0.05).

All the innate immune parameters investigated showed significant enhancement (p < 0.05) after feeding 3–6% SDP to the shrimp for 45 days, whereas those fed 1.5% SDP were not significantly different from the control (Fig 1). Specifically, the total hemocyte counts of the control, 1.5, 3, 4.5, and 6% SDP were 2.24, 2.40, 2.78, 2.85, and 2.93 × 10^6^ cells/mL, respectively (Fig 1A). The phagocytic activity was 62.67%, 63.50%, 73.83%, 74.67%, and 75.17%, respectively (Fig 1B). The phenoloxidase activity was 261.34, 265.12, 275.51, 276.13, and 275.54 units/min/mg of protein, respectively (Fig 1C). The superoxide dismutase (SOD) activity was 56.65%, 57.92%, 62.83%, 62.86%, and 63.85%, respectively (Fig 1D).

### Experiment 2: Effects of SDP on resistance to *V*. *parahaemolyticus* infection of shrimp after immersion challenge

There was no difference in the average weight gain between the *V*. *parahaemolyticus*-infected shrimp that were fed 1.5–6% SDP and the positive control on day 4 following the immersion challenge (Table 5). The weight gains of all these groups were significantly lower (p < 0.05) than those of the negative control shrimp. However, the great benefit of SDP supplementation was revealed by the survival rate. On day 4, the average survival rates of the shrimp that fed 3–6% SDP were at least 45%, significantly higher (p < 0.05) than those of the 1.5% SDP (32%) and the positive control (29%) (Table 5). No mortality was recorded in the negative control group.

**Table 5:** Effects of SDP on the weight gain and survival rate of the shrimp on day 4 after *Vibrio parahaemolyticus* immersion challenge.

Treatment group	Weight gain (g)	Survival rate (%)
**Negative control (without *V*. *parahaemolyticus*)**	0.27 ± 0.10^a^	100.00 ± 0.00^a^
**Positive control (with *V*. *parahaemolyticus*)**	0.05 ± 0.03^b^	29.17 ± 1.67^c^
**1.5% SDP**	0.05 ± 0.03^b^	31.67 ± 1.92^c^
**3% SDP**	0.10 ± 0.02^b^	45.00 ± 1.92^b^
**4.5% SDP**	0.08 ± 0.05^b^	45.00 ± 4.30^b^
**6% SDP**	0.08 ± 0.04^b^	48.33 ± 1.92^b^

The data was presented as mean ± SD. Means with different superscripts in a column are significantly different from each other (p < 0.05).

**Fig 2:**
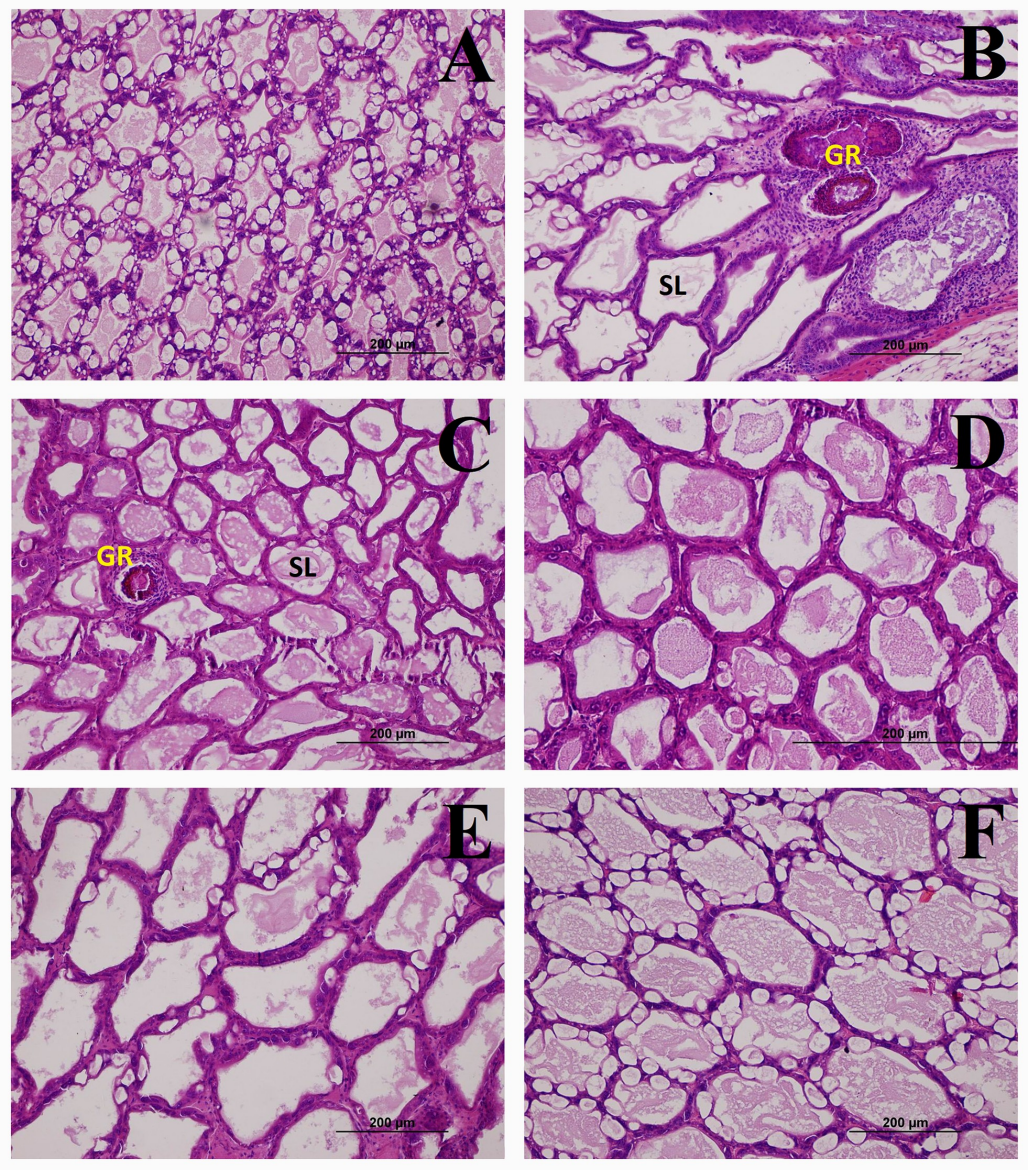
Effects of SDP on histopathology of the hepatopancreas of the *Vibrio parahaemolyticus*-infected shrimp. Hepatopancreas of the shrimp in the negative control (**A**), positive control (**B**), 1.5% SDP (**C**), 3% SDP (**D**), 4.5% SDP (**E**), and 6% SDP groups (**F**) on day 4 after immersion challenge with *Vibrio parahaemolyticus* (10^5^ CFU/mL) (H&E stain). GR, granulomatous encapsulation; SL, sloughing of the hepatopancreatic tubules.

The hepatopancreas of the *V*. *parahaemolyticus*-infected positive control and the 1.5% SDP group showed signs of bacterial infection including granulomatous encapsulation and sloughing of the hepatopancreatic tubules (Fig 2). The lower degree of pathology was seen in the other three SDP groups. No histopathological change was detected in the negative control shrimp.

## Discussion

The present study demonstrated that the shrimp feed supplemented with porcine SDP, especially at 3–6% of the diet, could improve the growth performance, survival, feed utilization, immune responses, and reduce the mortality caused by *V*. *parahaemolyticus* infection. The effective dose of SDP for improving the overall health status of the Pacific white shrimp was mainly in agreement with those reported in the pig studies (5–8% SDP) [7–11]. Multiple modes of action of SDP have been proposed. The positive results regarding the growth performance and feed utilization could be partly associated with the high nutritional value [3], high digestibility of SDP [15–17], improved intestinal morphology including increased villi height [7,10,12,20,29], and enhanced digestive enzyme activities [10]. In addition, some studies reported lower serum urea nitrogen of the SDP-fed pig, implying that the amino acid catabolism was reduced, and hence more dietary protein was utilized for growth [10,30].

However, it is widely accepted that health-promoting effect of SDP is mainly attributed to the immunomodulatory property of SDP on the intestinal barrier [3,6,31]. The anti-inflammatory activities of SDP including suppressing pro-inflammatory cytokine production (e.g., TNF-α and IFN-γ), stimulating anti-inflammatory cytokine production (e.g., IL-10 and TGF-β), inhibiting inflammatory cell infiltration in the gut mucosa and lamina propria, and improving intestinal barrier function are well-recognized especially in rodent models [32–37], and in weaned pigs to a lesser extent [8,38]. Continuous exposure of the intestinal mucosa to bacterial pathogens or toxins can cause chronic overstimulation of the mucosal immune system, thereby impairing intestinal barrier function and resulting in poor nutrient absorption. SDP administrations to rodents and pigs have been shown to reduce mucosal immunity over-activation which helps maintain the functional gut barrier and allows for more energy available for the growth as well as other productive function [3,6,31].

Although several biologically active compounds can be found in SDP [5], it is commonly believed that immunoglobulins (Ig), particularly IgG, are the main active component responsible for the observed growth-promoting activity of the SDP. This hypothesis has been supported by the fact that only the IgG-rich fraction resulted in increased weight gain and feed intake in weaning pigs, but neither the albumin-rich nor the low molecular weight fractions significantly affected growth performance [9]. Furthermore, dietary SDP and Ig concentrate which contained a similar amount of IgG (about 1% of diet) exhibited equivalent efficacy in reducing intestinal inflammation in rats [32,39]. *In vitro* studies demonstrated that animal IgG was able to bind to several Gram-positive and Gram-negative pathogenic bacteria or their components/products such as lipopolysaccharide (LPS) and staphylococcal enterotoxin B (SEB) [40–42]. Interestingly, ovine IgG was able to bind and suppress pathogenic bacteria of human origin with a varying degree of inhibition [40], suggesting that the binding between IgG and pathogens was not necessarily species-specific. Similarly, SDP from chicken blood [10,43] and cattle blood [9] did exhibit health benefits when fed to weaned pigs. In addition, serum bovine IgG has been applied in human gastrointestinal diseases, particularly HIV-associated enteropathy and diarrhea-prominent irritable bowel syndrome, with promising results [44,45]. Our findings which revealed that porcine SDP has positive effects on the health of Pacific white shrimp was also in agreement with these studies. The presence of cross-reactivity between the SDP or IgG sources and the animal recipients might imply that some IgG in the dietary SDP can bind to highly-conserved microbial antigens (such as LPS) and preventing the microbes from activating inflammatory responses [44]. Other functional components in SDP, besides IgG alone, like transferrin, bioactive peptides, growth factors, cytokines, and other compounds may also contribute to the health benefits observed in multiple species fed diets with SDP.

The anti-inflammatory activity of SDP was most well-studied in rodent models. Challenging mice and rats with SEB via intraperitoneal injection resulted in a strong intestinal inflammation as indicated by leukocyte infiltration, the release of inflammatory mediators, and increase permeability of the intestinal mucosa. SDP and IgG were shown to attenuate these changes by reducing the activated immune cells, pro-inflammatory cytokines, and intestinal barrier permeability [32,36,39,46]. In contrast to the modulated immune responses in the rodent models of inflammation, the current study found that SDP increased circulatory immune responses in shrimp. The reason why SDP possesses both immune-suppressing and immune-stimulating activities was not well-understood, but are possibly associated with a complex interplay between the site of action, IgG concentration, other bioactive substances in the SDP, immune status of the animals, and other factors. It should be mentioned that the immunostimulatory effect of SDP was reported in other related studies as well. For example, serum lysozyme, alternative complement pathway, and bactericidal activities of gilthead seabream fed 3–6% porcine SDP for 60 days were significantly increased compared to those fed 0% SDP [18]. Nile tilapia fed 6.63% porcine SDP for 60 days had a significantly higher leukocyte count than the control [20]. In one preliminary trial [22], kuruma shrimp (*Marsupenaeus japonicus*) fed 2–6% SDP for 7 days showed enhanced phagocytosis and phenoloxidase activity, which were generally consistent with our results.

In the current study, the survival rates of the SDP-fed shrimp at the end of both Experiment 1 and 2 were significantly higher than the control shrimp. The elevated immune responses might partially but not solely contribute to the desired outcome. Another sensible explanation might be due to the ability of IgG to bind certain bacterial components like LPS which ultimately lead to bacterial inhibition [40,42]. Given that LPS is presented in the cell wall of all Gram-negative bacteria including *V*. *parahaemolyticus*, a similar reaction was likely applied in our case as well although the antimicrobial activity of the SDP or IgG against the challenged strain of *V*. *parahaemolyticus* was not determined. The lower degree of histopathological changes in the hepatopancreas of SDP-fed shrimp supports the protective action of SDP from bacterial infection. Unsurprisingly, the benefit of SDP in improving the survival rate was more prominent in the *V*. *parahaemolyticus*-challenged shrimp (Experiment 2) than the healthy shrimp (Experiment 1). Greater efficacy of SDP also observed in the pigs reared in poor hygiene conditions compared to a cleaner environment [47], supporting the speculation that the mode of action of SDP might be related to the anti-pathogen and anti-inflammatory activities. Finally, it is worth mentioning that orally administered IgG can survive passage through the mammalian gastrointestinal tract without being absorbed or loss of their biological activities [48], and examples of dietary SDP or IgG in reducing diseases severity [49,50] and pathogen shedding [41,51] in experimental challenged animals are not uncommon. In short, our study proved that porcine SDP, along with other health-promoting agents such as herbal extracts, organic acids, carotenoids, and probiotics [1,2,52], can improve the health conditions and enhance the disease resistance of aquatic animals, thereby helping to reduce the unnecessary uses of antibiotics in aquaculture.

## Conclusions

The health benefit of applying porcine SDP in the shrimp feed was evident in the present study, especially at the inclusion level of 4.5–6% diet (45–60 g/kg diet). The SDP was shown to improve growth performance, survival, feed utilization, immune responses, and reduce the mortality of *V*. *parahaemolyticus*-infected Pacific white shrimp. Therefore, it could potentially be applied in shrimp farming as an alternative to antibiotics.

## Supporting information

S1 Fig The average survival rate of the shrimp after *Vibrio parahaemolyticus* immersion challenge. (TIFF)

S1 Table Effects of SDP on body weight (Experiment 1). (DOCX)

S2 Table Effects of SDP on feed conversion ratio (FCR), feed efficiency (FE), and protein efficiency ratio (PER) (Experiment 1). (DOCX)

S3 Table Effects of SDP on survival rate (Experiment 1). (DOCX)

S4 Table Effects SDP on immune parameters of healthy shrimp (Experiment 1). (DOCX)

S5 Table Effects of SDP on the weight gain and survival rate of the shrimp on day 4 after *Vibrio parahaemolyticus* immersion challenge (Experiment 2). (DOCX)
