# Supplementary figures and images for: Effects of spray-dried animal plasma on growth performance, survival, feed utilization, immune responses, and resistance to Vibrio parahaemolyticus infection of Pacific white shrimp (Litopenaeus vannamei)
Source: PLoS One. 2021 Sep 24;16(9):e0257792. doi: 10.1371/journal.pone.0257792 (PMC8462686; doi:10.1371/journal.pone.0257792)

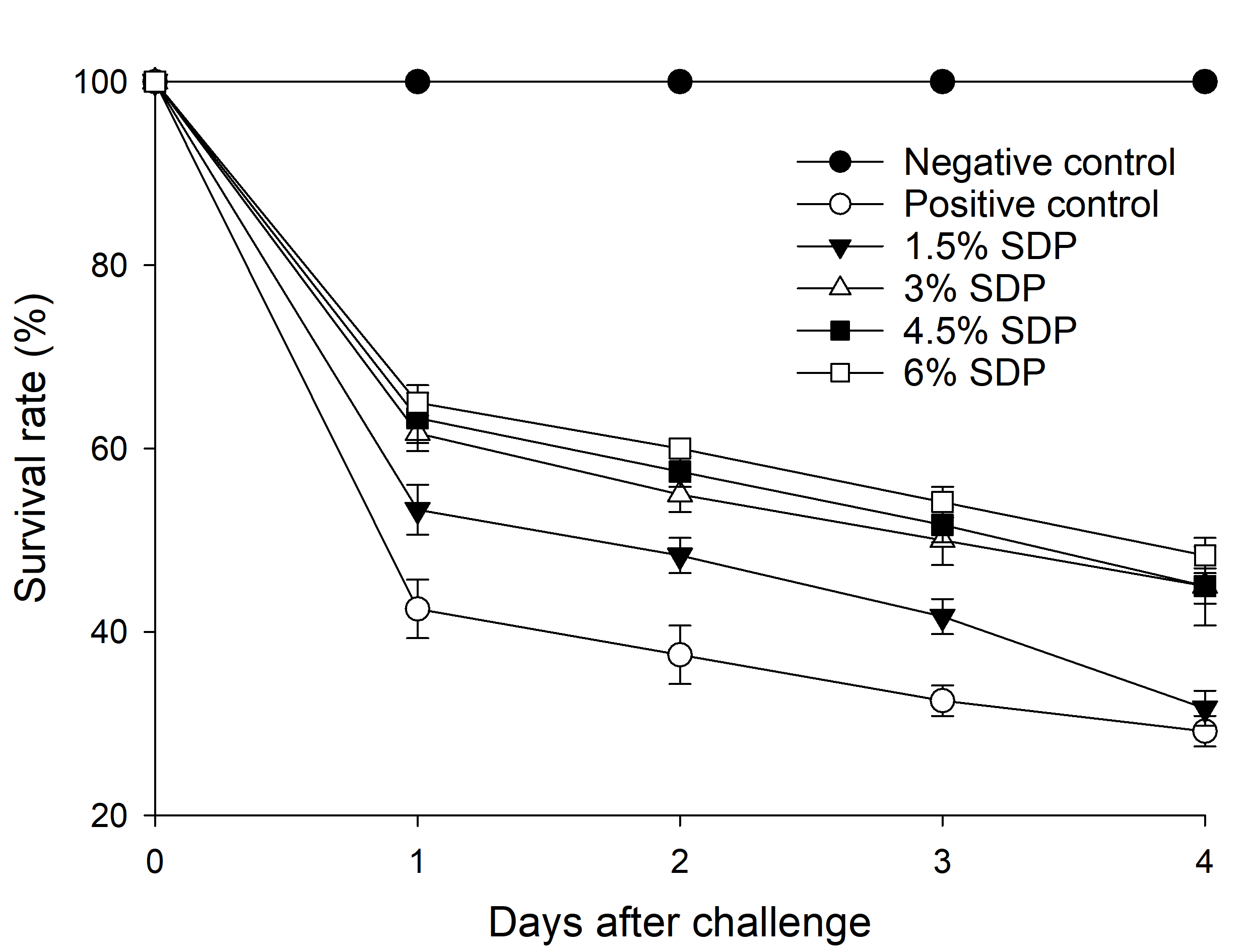

Supplement: S1 Fig — (TIFF) [file pone.0257792.s001.tiff]
