# Supplementary material for: Effects of spray-dried animal plasma on growth performance, survival, feed utilization, immune responses, and resistance to Vibrio parahaemolyticus infection of Pacific white shrimp (Litopenaeus vannamei)
Source: PLoS One. 2021 Sep 24;16(9):e0257792. doi: 10.1371/journal.pone.0257792 (PMC8462686; doi:10.1371/journal.pone.0257792)
Supplement: S1 Table — (DOCX) [file pone.0257792.s002.docx]

**Supporting information**

**Effects of spray-dried animal plasma on growth performance, survival, feed utilization, immune responses, and resistance to *Vibrio parahaemolyticus* infection of Pacific white shrimp (*Litopenaeus vannamei*)**

**Table S1. Effects of SDP on body weight (Experiment 1)**

| **Treatment** | **Body weight (g) at day 30** | | **Body weight (g) at day 45** | |
| --- | --- | --- | --- | --- |
|  | **Raw data** | **mean ± SD** | **Raw data** | **mean ± SD** |
| **Control 1** | 1.13 | 1.10 ± 0.03^a^ | 2.59 | 2.58 ± 0.02^c^ |
| **Control 2** | 1.06 |  | 2.55 |  |
| **Control 3** | 1.10 |  | 2.56 |  |
| **Control 4** | 1.12 |  | 2.61 |  |
| **1.5% SDP 1** | 1.15 | 1.14 ± 0.02^b^ | 2.86 | 2.73 ± 0.12^c^ |
| **1.5% SDP 2** | 1.12 |  | 2.73 |  |
| **1.5% SDP 3** | 1.12 |  | 2.74 |  |
| **1.5% SDP 4** | 1.15 |  | 2.57 |  |
| **3% SDP 1** | 1.22 | 1.20 ± 0.03^a^ | 2.98 | 2.88 ± 0.10^b^ |
| **3% SDP 2** | 1.23 |  | 2.78 |  |
| **3% SDP 3** | 1.20 |  | 2.82 |  |
| **3% SDP 4** | 1.16 |  | 2.94 |  |
| **4.5% SDP 1** | 1.22 | 1.21 ± 0.01^a^ | 2.97 | 3.06 ± 0.14^a^ |
| **4.5% SDP 2** | 1.20 |  | 2.95 |  |
| **4.5% SDP 3** | 1.20 |  | 3.08 |  |
| **4.5% SDP 4** | 1.22 |  | 3.26 |  |
| **6% SDP 1** | 1.27 | 1.23 ± 0.04^a^ | 3.16 | 3.13 ± 0.07^a^ |
| **6% SDP 2** | 1.23 |  | 3.12 |  |
| **6% SDP 3** | 1.18 |  | 3.19 |  |
| **6% SDP 4** | 1.26 |  | 3.04 |  |

The data was presented as mean ± SD. Means with different superscripts in a column are significantly different from each other (p < 0.05).
