# Supplementary material for: Effects of spray-dried animal plasma on growth performance, survival, feed utilization, immune responses, and resistance to Vibrio parahaemolyticus infection of Pacific white shrimp (Litopenaeus vannamei)
Source: PLoS One. 2021 Sep 24;16(9):e0257792. doi: 10.1371/journal.pone.0257792 (PMC8462686; doi:10.1371/journal.pone.0257792)
Supplement: S2 Table — (DOCX) [file pone.0257792.s003.docx]

**Table S2. Effects of SDP on feed conversion ratio (FCR), feed efficiency (FE), and protein efficiency ratio (PER) (Experiment 1)**

| **Treatment** | **FCR** | | **FE** | | **PER** | |
| --- | --- | --- | --- | --- | --- | --- |
|  | **Raw data** | **mean ± SD** | **Raw data** | **mean ± SD** | **Raw data** | **mean ± SD** |
| **Control 1** | 1.56 | 1.55 ± 0.02^b^ | 64.23 | 64.44 ± 0.90^b^ | 1.65 | 1.66 ± 0.02^c^ |
| **Control 2** | 1.55 |  | 64.34 |  | 1.66 |  |
| **Control 3** | 1.57 |  | 63.51 |  | 1.64 |  |
| **Control 4** | 1.52 |  | 65.67 |  | 1.69 |  |
| **1.5% SDP 1** | 1.45 | 1.54 ± 0.08^b^ | 68.95 | 64.86 ± 3.38^b^ | 1.78 | 1.67 ± 0.09^bc^ |
| **1.5% SDP 2** | 1.52 |  | 65.65 |  | 1.69 |  |
| **1.5% SDP 3** | 1.56 |  | 64.03 |  | 1.65 |  |
| **1.5% SDP 4** | 1.64 |  | 60.82 |  | 1.57 |  |
| **3% SDP 1** | 1.39 | 1.45 ± 0.05^a^ | 71.81 | 69.11 ± 2.59^a^ | 1.85 | 1.78 ± 0.07^ab^ |
| **3% SDP 2** | 1.50 |  | 66.85 |  | 1.72 |  |
| **3% SDP 3** | 1.49 |  | 66.94 |  | 1.72 |  |
| **3% SDP 4** | 1.41 |  | 70.85 |  | 1.83 |  |
| **4.5% SDP 1** | 1.42 | 1.43 ± 0.08^a^ | 70.34 | 70.24 ± 3.81^a^ | 1.81 | 1.81 ± 0.10^a^ |
| **4.5% SDP 2** | 1.52 |  | 65.77 |  | 1.69 |  |
| **4.5% SDP 3** | 1.43 |  | 69.77 |  | 1.80 |  |
| **4.5% SDP 4** | 1.33 |  | 75.07 |  | 1.93 |  |
| **6% SDP 1** | 1.39 | 1.39 ± 0.05^a^ | 71.72 | 71.95 ± 2.45^a^ | 1.85 | 1.85 ± 0.06^a^ |
| **6% SDP 2** | 1.34 |  | 74.86 |  | 1.93 |  |
| **6% SDP 3** | 1.38 |  | 72.33 |  | 1.86 |  |
| **6% SDP 4** | 1.45 |  | 68.88 |  | 1.77 |  |

The data was presented as mean ± SD. Means with different superscripts in a column are significantly different from each other (p < 0.05).
