# Supplementary material for: Effects of spray-dried animal plasma on growth performance, survival, feed utilization, immune responses, and resistance to Vibrio parahaemolyticus infection of Pacific white shrimp (Litopenaeus vannamei)
Source: PLoS One. 2021 Sep 24;16(9):e0257792. doi: 10.1371/journal.pone.0257792 (PMC8462686; doi:10.1371/journal.pone.0257792)
Supplement: S3 Table — (DOCX) [file pone.0257792.s004.docx]

**Table S3. Effects of SDP on survival rate (Experiment 1)**

| **Treatment** | **Survival rate (%) at day 20** | | **Survival rate (%) at day 30** | | **Survival rate (%) at day 45** | |
| --- | --- | --- | --- | --- | --- | --- |
|  | **Raw data** | **mean ± SD** | **Raw data** | **mean ± SD** | **Raw data** | **mean ± SD** |
| **Control 1** | 90.00 | 89.38 ± 5.99^a^ | 82.50 | 83.13 ± 0.72^b^ | 77.50 | 78.13 ± 0.72^b^ |
| **Control 2** | 86.25 |  | 83.75 |  | 78.75 |  |
| **Control 3** | 83.75 |  | 83.75 |  | 77.50 |  |
| **Control 4** | 97.50 |  | 82.50 |  | 78.75 |  |
| **1.5% SDP 1** | 86.25 | 86.56 ± 4.61^a^ | 86.25 | 83.44 ± 3.29^b^ | 81.25 | 80.31 ± 1.20^ab^ |
| **1.5% SDP 2** | 86.25 |  | 86.25 |  | 81.25 |  |
| **1.5% SDP 3** | 92.50 |  | 80.00 |  | 78.75 |  |
| **1.5% SDP 4** | 81.25 |  | 81.25 |  | 80.00 |  |
| **3% SDP 1** | 90.00 | 89.69 ± 1.57^a^ | 82.50 | 84.69 ± 3.87^ab^ | 81.25 | 80.94 ± 0.63^ab^ |
| **3% SDP 2** | 87.50 |  | 85.00 |  | 81.25 |  |
| **3% SDP 3** | 90.00 |  | 81.25 |  | 80.00 |  |
| **3% SDP 4** | 91.25 |  | 90.00 |  | 81.25 |  |
| **4.5% SDP 1** | 90.00 | 88.13 ± 3.31^a^ | 90.00 | 87.50 ± 2.70^ab^ | 88.75 | 85.94 ± 2.13^a^ |
| **4.5% SDP 2** | 83.75 |  | 83.75 |  | 83.75 |  |
| **4.5% SDP 3** | 91.25 |  | 88.75 |  | 85.00 |  |
| **4.5% SDP 4** | 87.50 |  | 87.50 |  | 86.25 |  |
| **6% SDP 1** | 86.25 | 92.50 ± 4.45^a^ | 86.25 | 88.75 ± 4.21^a^ | 85.00 | 86.25 ± 2.50^a^ |
| **6% SDP 2** | 96.25 |  | 95.00 |  | 90.00 |  |
| **6% SDP 3** | 92.50 |  | 87.50 |  | 85.00 |  |
| **6% SDP 4** | 95.00 |  | 86.25 |  | 85.00 |  |

The data was presented as mean ± SD. Means with different superscripts in a column are significantly different from each other (p < 0.05).
