# Supplementary material for: Effects of spray-dried animal plasma on growth performance, survival, feed utilization, immune responses, and resistance to Vibrio parahaemolyticus infection of Pacific white shrimp (Litopenaeus vannamei)
Source: PLoS One. 2021 Sep 24;16(9):e0257792. doi: 10.1371/journal.pone.0257792 (PMC8462686; doi:10.1371/journal.pone.0257792)
Supplement: S4 Table — (DOCX) [file pone.0257792.s005.docx]

**Table S4. Effects SDP on immune parameters of healthy shrimp (Experiment 1)**

| **Treatment** | **Total hemocyte count (10^6^ cells/mL)** | | **Phagocytic activity (%)** | | **Phenoloxidase activity (unit/min/mg of protein)** | | **SOD activity (% inhibition)** | |
| --- | --- | --- | --- | --- | --- | --- | --- | --- |
|  | **Raw data** | **mean ± SD** | **Raw data** | **mean ± SD** | **Raw data** | **mean ± SD** | **Raw data** | **mean ± SD** |
| **Control 1** | 2.15 | 2.24 ± 0.22^b^ | 63.33 | 62.67 ± 0.94^b^ | 252.38 | 261.34 ± 6.07^b^ | 56.77 | 56.65 ± 0.20^b^ |
| **Control 2** | 1.98 |  | 62.67 |  | 265.83 |  | 56.87 |  |
| **Control 3** | 2.38 |  | 61.33 |  | 263.33 |  | 56.50 |  |
| **Control 4** | 2.45 |  | 63.33 |  | 263.81 |  | 56.45 |  |
| **1.5% SDP 1** | 2.48 | 2.40 ± 0.10^b^ | 62.00 | 63.50 ± 2.20^b^ | 258.69 | 265.12 ± 9.37^b^ | 56.28 | 57.92 ± 2.89^b^ |
| **1.5% SDP 2** | 2.45 |  | 61.33 |  | 261.07 |  | 55.19 |  |
| **1.5% SDP 3** | 2.43 |  | 66.00 |  | 261.67 |  | 58.47 |  |
| **1.5% SDP 4** | 2.25 |  | 64.67 |  | 279.05 |  | 61.75 |  |
| **3% SDP 1** | 2.83 | 2.78 ± 0.04^a^ | 74.67 | 73.83 ± 1.26^a^ | 275.60 | 275.51 ± 1.95^a^ | 64.02 | 62.83 ± 1.39^a^ |
| **3% SDP 2** | 2.78 |  | 74.67 |  | 277.74 |  | 61.63 |  |
| **3% SDP 3** | 2.73 |  | 72.00 |  | 275.71 |  | 61.64 |  |
| **3% SDP 4** | 2.78 |  | 74.00 |  | 272.98 |  | 64.04 |  |
| **4.5% SDP 1** | 2.83 | 2.85 ± 0.05^a^ | 76.00 | 74.67 ± 1.44^a^ | 272.50 | 276.13 ± 2.78^a^ | 61.51 | 62.86 ± 1.08^a^ |
| **4.5% SDP 2** | 2.93 |  | 74.67 |  | 279.05 |  | 62.82 |  |
| **4.5% SDP 3** | 2.85 |  | 72.67 |  | 277.26 |  | 62.95 |  |
| **4.5% SDP 4** | 2.80 |  | 75.33 |  | 275.71 |  | 64.15 |  |
| **6% SDP 1** | 2.93 | 2.93 ± 0.08^a^ | 74.00 | 75.17 ± 1.84^a^ | 273.57 | 275.54 ± 2.66^a^ | 64.03 | 63.85 ± 0.50^a^ |
| **6% SDP 2** | 2.88 |  | 76.00 |  | 275.12 |  | 64.21 |  |
| **6% SDP 3** | 2.88 |  | 73.33 |  | 279.40 |  | 63.11 |  |
| **6% SDP 4** | 3.05 |  | 77.33 |  | 274.05 |  | 64.04 |  |

The data was presented as mean ± SD. Means with different superscripts in a column are significantly different from each other (p < 0.05).
