# Supplementary material for: Effects of spray-dried animal plasma on growth performance, survival, feed utilization, immune responses, and resistance to Vibrio parahaemolyticus infection of Pacific white shrimp (Litopenaeus vannamei)
Source: PLoS One. 2021 Sep 24;16(9):e0257792. doi: 10.1371/journal.pone.0257792 (PMC8462686; doi:10.1371/journal.pone.0257792)
Supplement: S5 Table — (DOCX) [file pone.0257792.s006.docx]

**Table S5. Effects of SDP on the weight gain and survival rate of the shrimp on day 4 after *Vibrio parahaemolyticus* immersion challenge (Experiment 2)**

| **Treatment** | **Weight gain (g)** | | **Survival rate (%)** | |
| --- | --- | --- | --- | --- |
|  | **Raw data** | **mean ± SD** | **Raw data** | **mean ± SD** |
| **Neg. Control 1** | 0.15 | 0.27 ± 0.10^a^ | 100.00 | 100.00 ± 0.00^a^ |
| **Neg. Control 2** | 0.30 |  | 100.00 |  |
| **Neg. Control 3** | 0.25 |  | 100.00 |  |
| **Neg. Control 4** | 0.38 |  | 100.00 |  |
| **Pos. Control 1** | 0.03 | 0.05 ± 0.03^b^ | 26.67 | 29.17 ± 1.67^c^ |
| **Pos. Control 2** | 0.04 |  | 30.00 |  |
| **Pos. Control 3** | 0.10 |  | 30.00 |  |
| **Pos. Control 4** | 0.04 |  | 30.00 |  |
| **1.5% SDP 1** | 0.03 | 0.05 ± 0.03^b^ | 33.33 | 31.67 ± 1.92^c^ |
| **1.5% SDP 2** | 0.09 |  | 33.33 |  |
| **1.5% SDP 3** | 0.04 |  | 30.00 |  |
| **1.5% SDP 4** | 0.04 |  | 30.00 |  |
| **3% SDP 1** | 0.07 | 0.10 ± 0.02^b^ | 43.33 | 45.00 ± 1.92^b^ |
| **3% SDP 2** | 0.11 |  | 43.33 |  |
| **3% SDP 3** | 0.12 |  | 46.67 |  |
| **3% SDP 4** | 0.10 |  | 46.67 |  |
| **4.5% SDP 1** | 0.05 | 0.08 ± 0.05^b^ | 43.33 | 45.00 ± 4.30^b^ |
| **4.5% SDP 2** | 0.15 |  | 40.00 |  |
| **4.5% SDP 3** | 0.05 |  | 46.67 |  |
| **4.5% SDP 4** | 0.05 |  | 50.00 |  |
| **6% SDP 1** | 0.04 | 0.08 ± 0.04^b^ | 46.67 | 48.33 ± 1.92^b^ |
| **6% SDP 2** | 0.07 |  | 46.67 |  |
| **6% SDP 3** | 0.07 |  | 50.00 |  |
| **6% SDP 4** | 0.13 |  | 50.00 |  |

The data was presented as mean ± SD. Means with different superscripts in a column are significantly different from each other (p < 0.05).
